# Supplementary material for: First look by the Yutu-2 rover at the deep subsurface structure at the lunar farside
Source: Nat Commun. 2020 Jul 9;11:3426. doi: 10.1038/s41467-020-17262-w (PMC7347897; doi:10.1038/s41467-020-17262-w)
Supplement: Supplementary file 1 — Supplementary Information [file 41467_2020_17262_MOESM1_ESM.pdf]

Supporting Information for

First Look by the Yutu-2 Rover at the Deep Subsurface Structure at the Lunar Farside

by Lai et al.

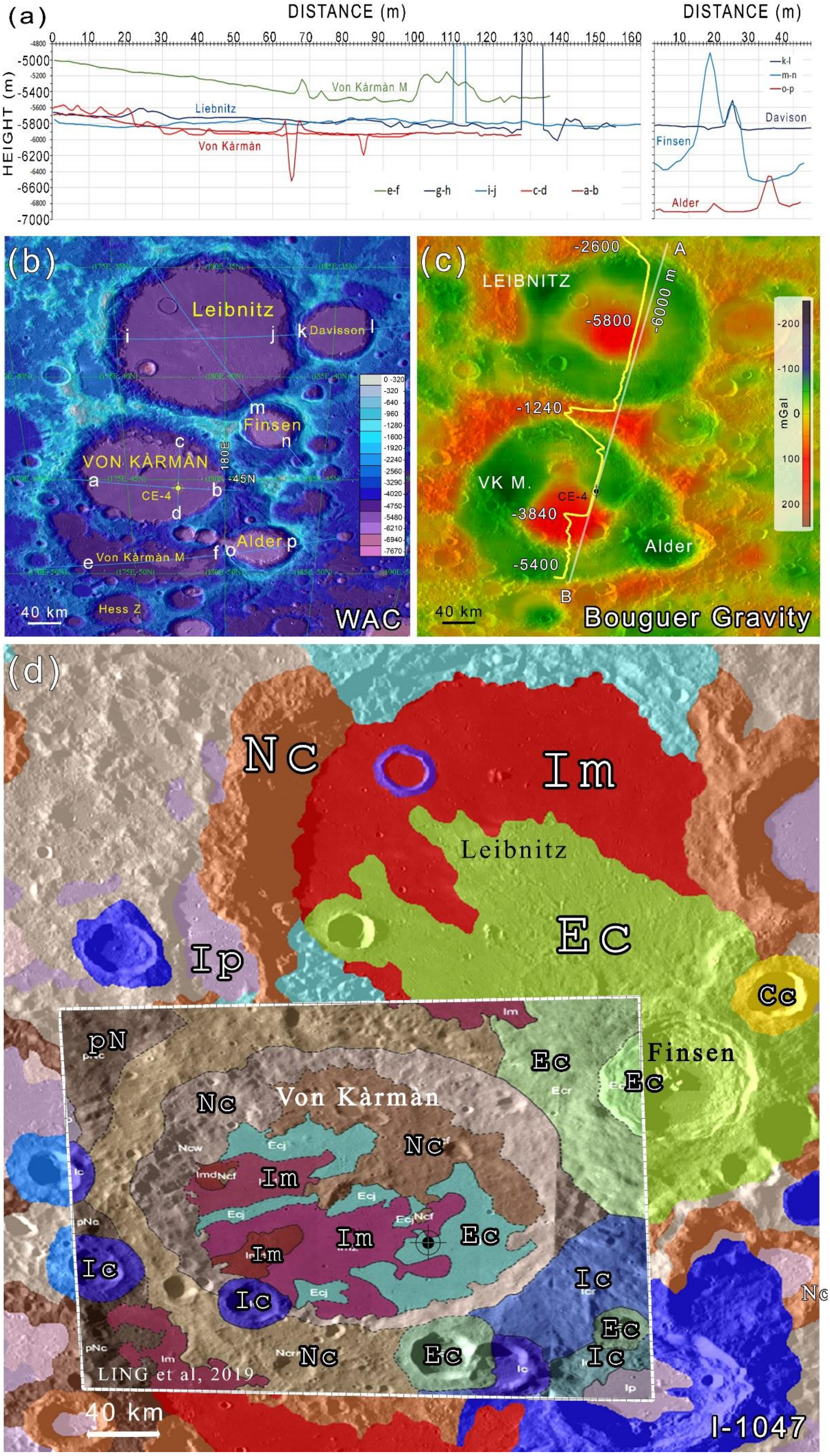

(a)
DISTANCE (m)
HEIGHT (m)
Von Kàrmàn M
Liebnitz
Von Kàrmàn
e-f
g-h
i-j
c-d
a-b
DISTANCE (m)
k-l
m-n
o-p
Davison
Finsen
Alder
(b)
Leibnitz
Davisson
Finsen
VON KÀRMÀN
180E
-45N
CE-4
Alder
Von Kàrmàn M
Hess Z
40 km
WAC
(c)
LEIBNITZ
-2600
-5800
-6000 m
A
-1240
VK M.
CE-4
-3840
-5400
Alder
B
mGal
40 km
Bouguer Gravity
(d)
Nc
Im
Leibnitz
Ec
Ip
Cc
pN
Von Kàrmàn
Finsen
Ic
LING et al, 2019
40 km
I-1047

Supplementary Figure 1: Geologic settings of Chang'e-4 (CE-4) landing site. (a) Cross-sections topography data[6] (SLDEM2015+LOLA) above (b), the corresponding high-resolution lunar elevation map; (c) Bouguer Gravity map (deg. 60 to 600)[7], including elevation profile for A-B; (d) 2019 map of the landing site superimposed on the Wilhelms I-1047 geologic map [1,8,9]; nomenclatures of geological units relate to the lunar chronology system (Copernican 'C', Eratosthenian 'E', Imbrian 'I', Nectarian 'N', and pre-Nectarian 'pN', followed by description of materials (crater 'c' or mare 'm') and terminated by map's authors sub-classes classification (please refer to original maps for full descriptions and nomenclatures): All maps are draped over a Wide Angle Camera (WAC) global morphologic base map.

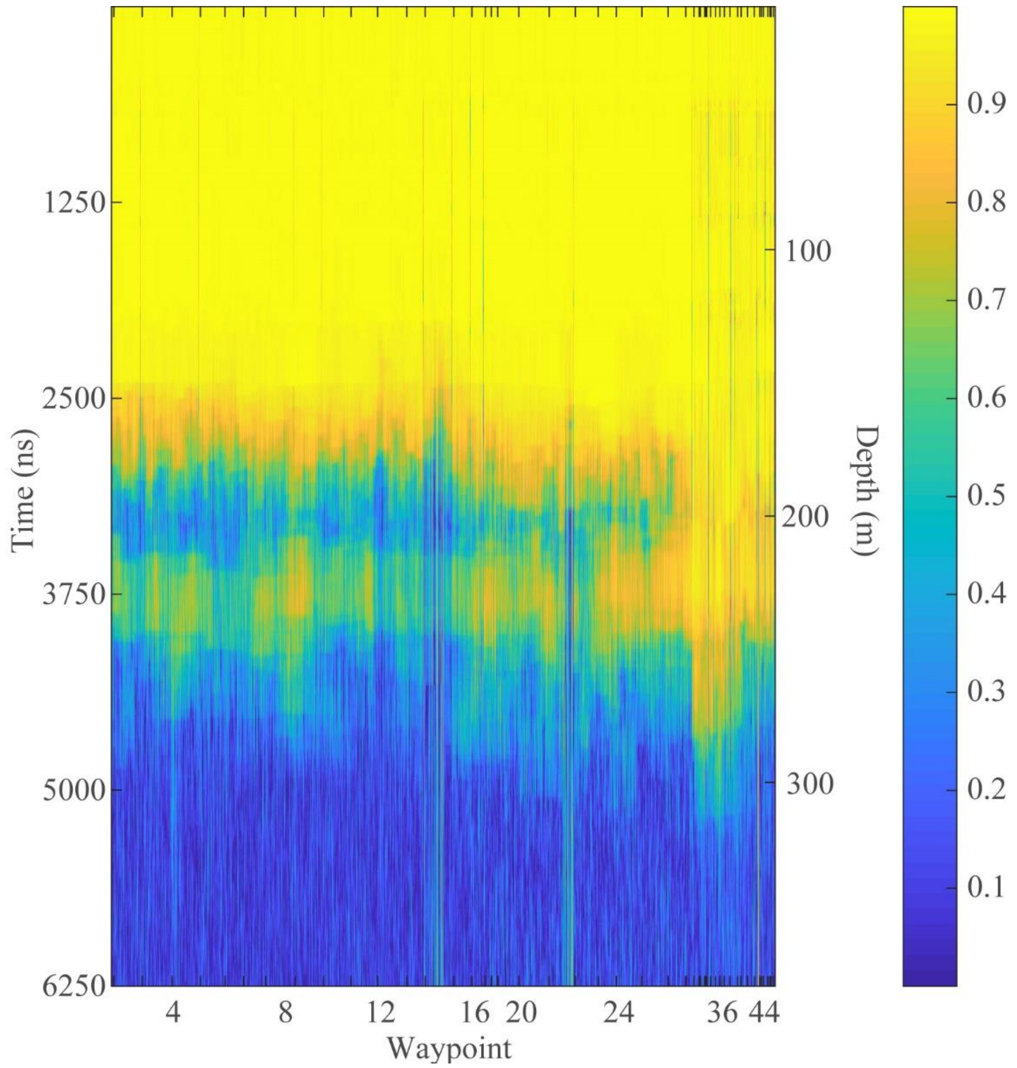

Supplementary Figure 2: The estimated penetration depth of CE-4 LPR. Color-scale is a correlation factor and is unitless.

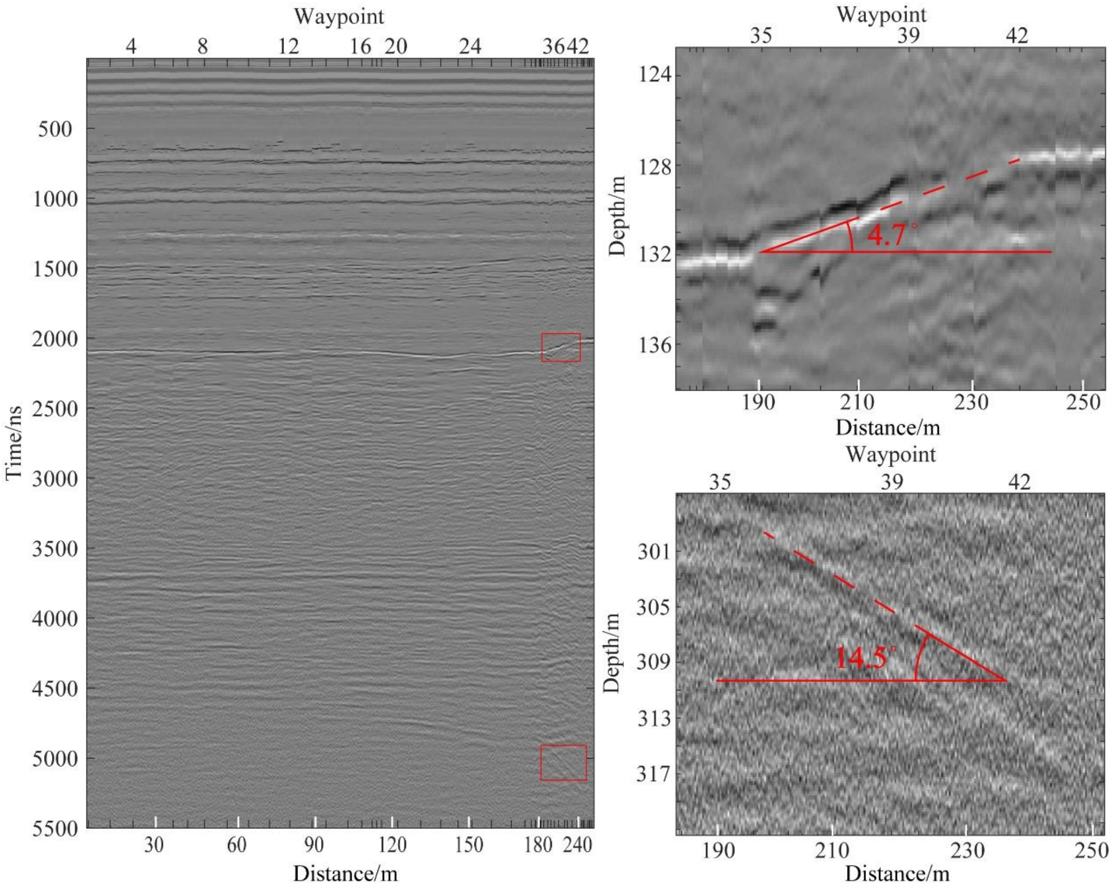

Supplementary Figure 3: The slope calculation of the reflectors.

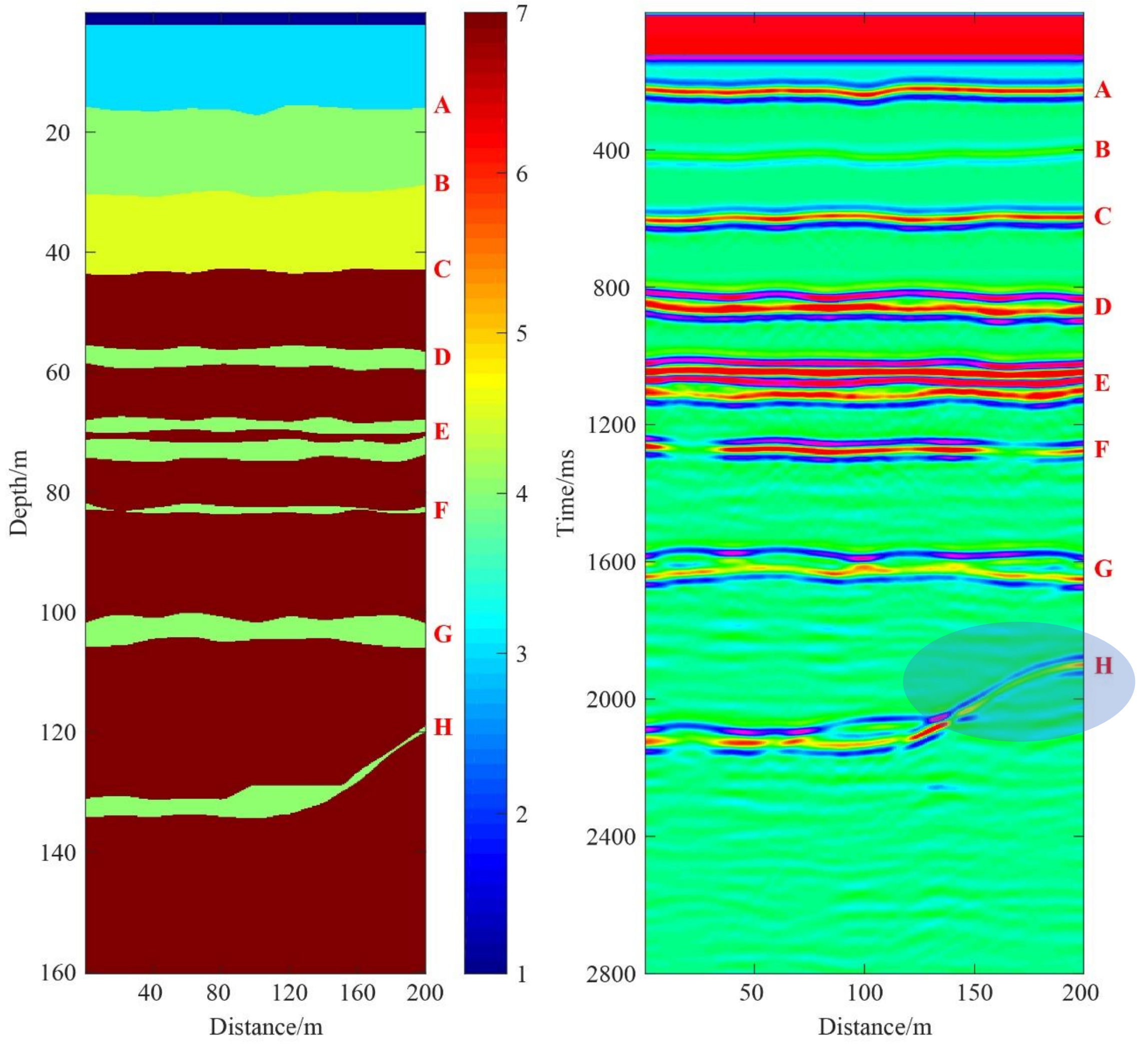

Supplementary Figure 4: The simulation results of the case of the rim of a buried crater. The left figure is the model used for the 2D finite-difference time-domain (FDTD) method. The uprising end of H represents the rim part of a small crater. The color bar indicates different permittivity values used for layers at different depths. The right figure shows the simulation results. Obvious reflectors occur at each interface and the reflector H upraises at the right end, similar to the LPR results shown in Figure 1 (a) (b).

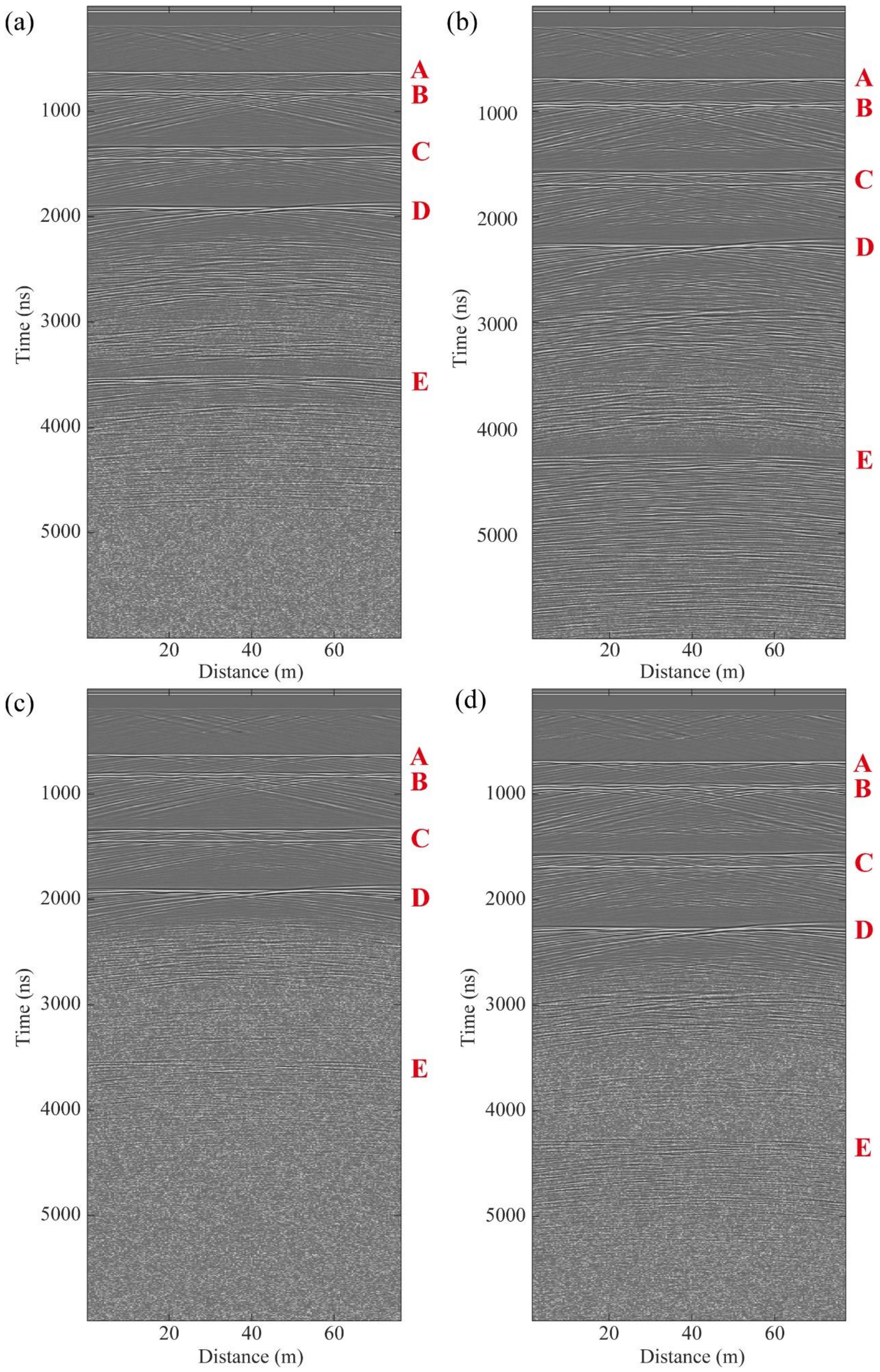

Supplementary Figure 5: The LPR simulation results with different dielectric permittivity and loss tangent values of mare basalt. The LPR simulation results of (a)

ε(mare basalt)= 6.5 and $\tan\delta = 0.005$, (b) ε(mare basalt)= 10 and $\tan\delta = 0.005$, (c) ε(mare basalt)= 6.5 and $\tan\delta = 0.009$, (d) ε(mare basalt)= 10 and $\tan\delta = 0.009$.

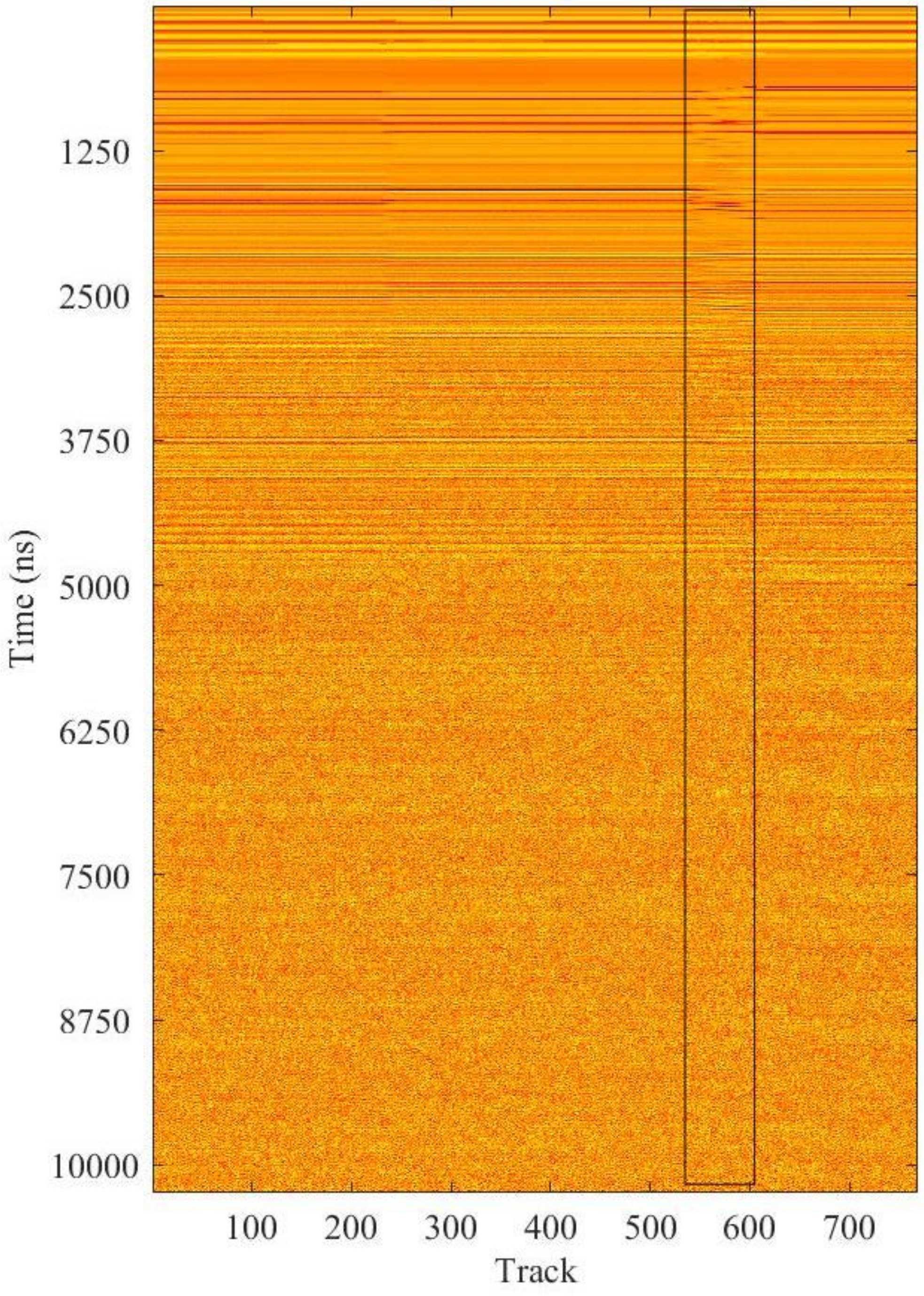

Supplementary Figure 6: An example of repetitive data removal. The rover may stop to perform other scientific measurements when LPR is on, so LPR collected data at same location repetitively. We only keep the average value of repeated data and motion data indicated by the black frame. File ID: CE4_GRAS_LPR-1_SCI_N_20190209130801_20190210033400_0009_A.2B

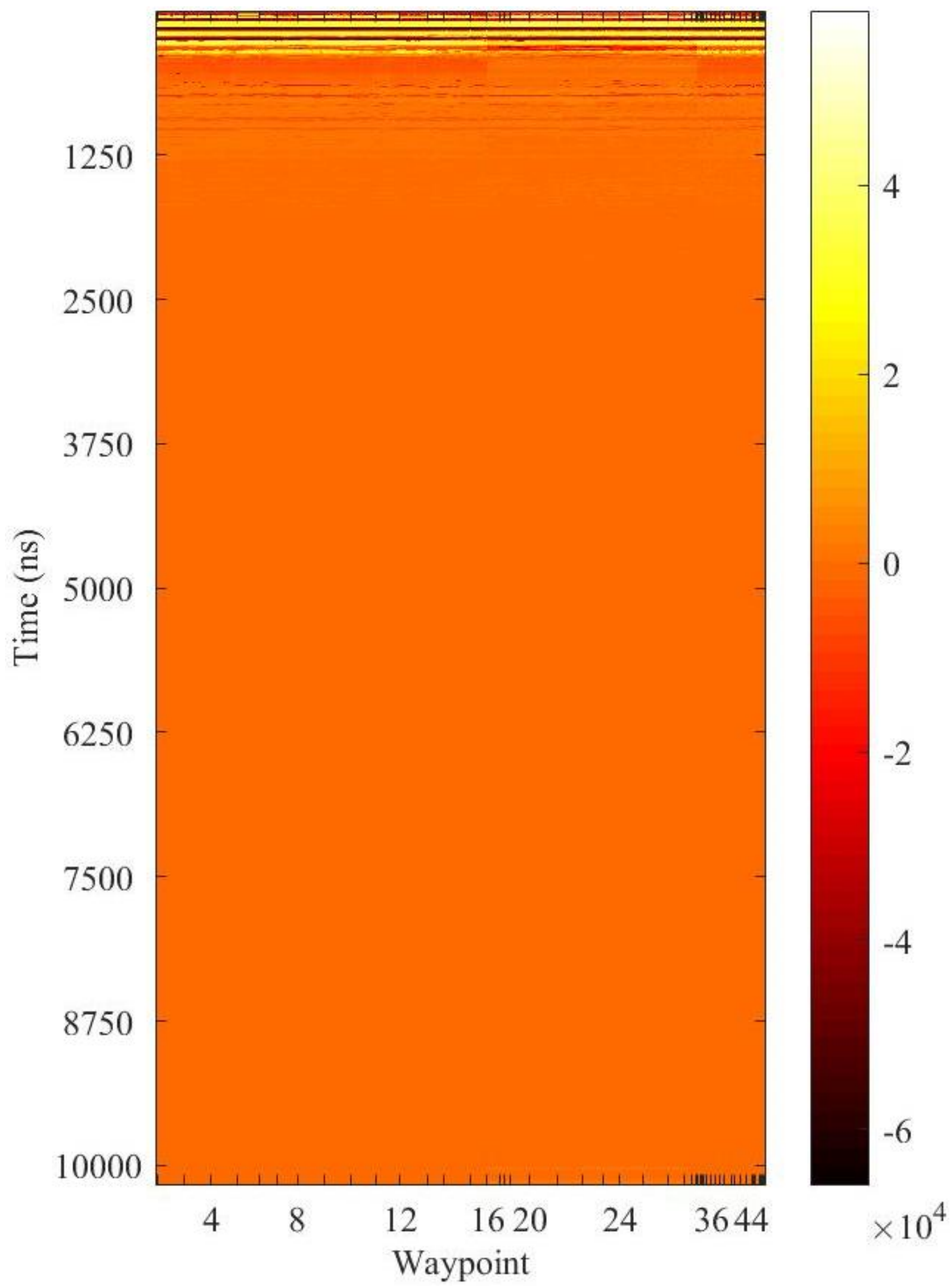

Supplementary Figure 7: Assembled nine-month LPR data. Assembled all the LPR motion data with 38 data files in Table S1.

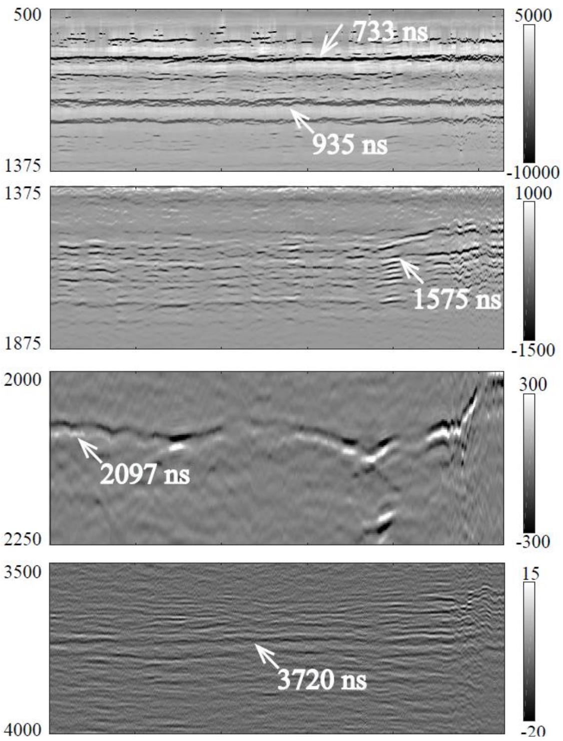

Supplementary Figure 8: Identified radar features at CE-4 site. Identified radar features from CE-4 LPR results. Y axis indicates the depth of the recognized radar features in ns.

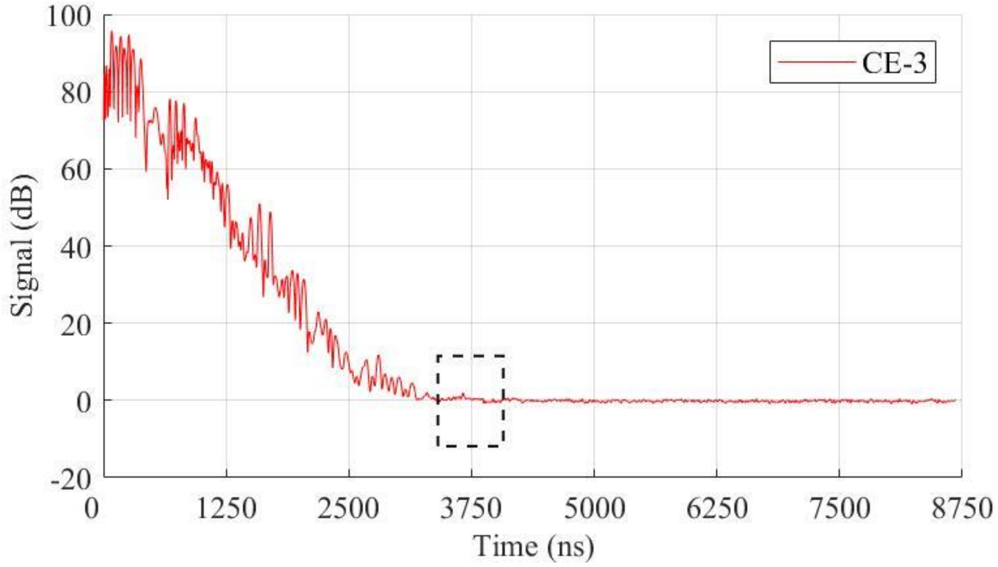

(a)

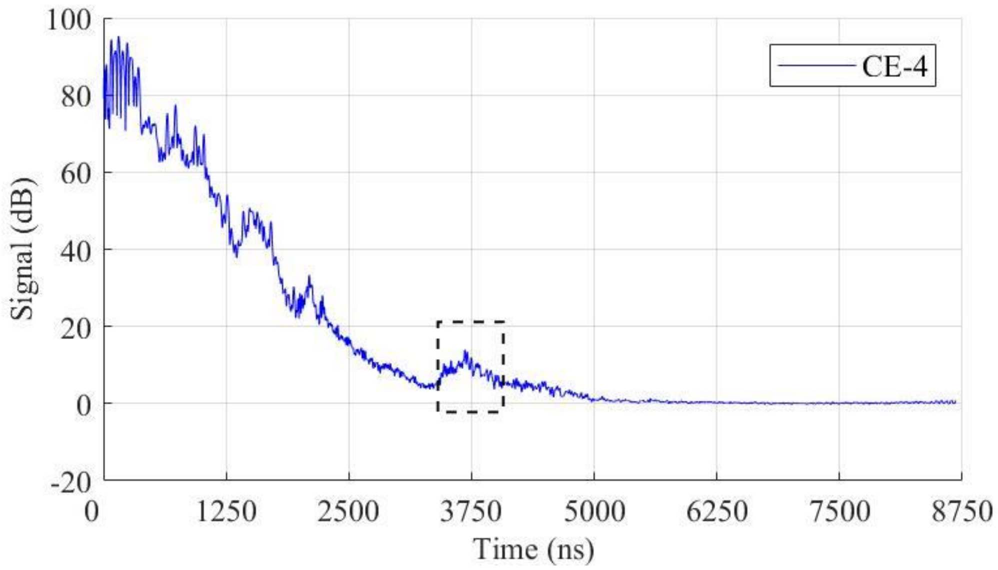

(b)

Supplementary Figure 9: The aggregated LPR CH-1 data tracks at CE-3 and CE-4 sites. The aggregated LPR CH-1 data tracks at (a) CE-3 site (b) CE-4 site.

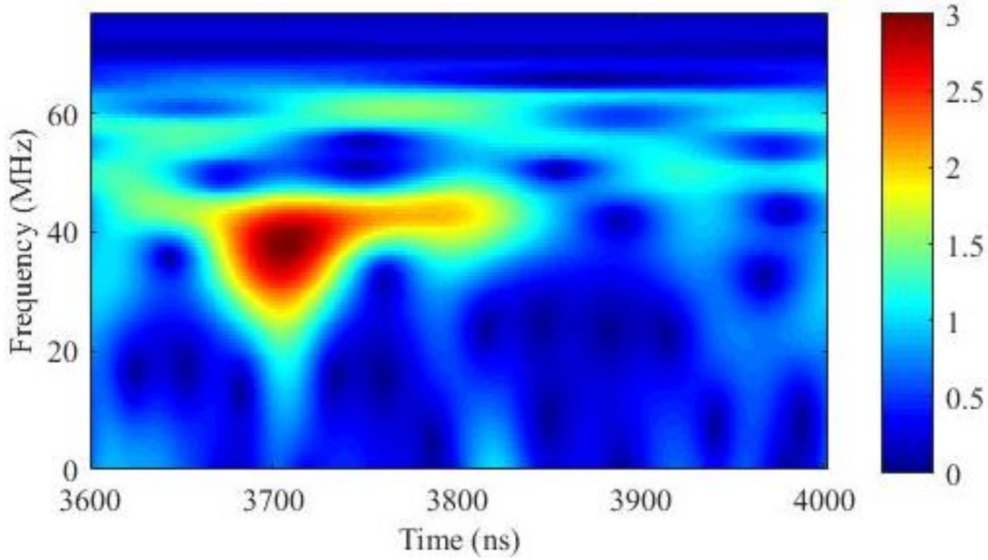

Supplementary Figure 10: The S-transform results of LPR CH-1 at CE-4 site.

Supplementary Table 1: File IDs of used Chang'E-4 LPR data

index	Filename
1	CE4_GRAS_LPR-1_SCI_N_20190104004000_20190109213900_0001_A.2B
2	CE4_GRAS_LPR-1_SCI_N_20190109213901_20190111150200_0002_A.2B
3	CE4_GRAS_LPR-1_SCI_N_20190111150201_20190112102100_0003_A.2B
4	CE4_GRAS_LPR-1_SCI_N_20190131080301_20190201054300_0005_A.2B
5	CE4_GRAS_LPR-1_SCI_N_20190201054301_20190208101200_0006_A.2B
6	CE4_GRAS_LPR-1_SCI_N_20190208101201_20190209053100_0007_A.2B
7	CE4_GRAS_LPR-1_SCI_N_20190209053101_20190209130800_0008_A.2B
8	CE4_GRAS_LPR-1_SCI_N_20190209130801_20190210033400_0009_A.2B
9	CE4_GRAS_LPR-1_SCI_N_20190210033401_20190211100800_0010_A.2B
10	CE4_GRAS_LPR-1_SCI_N_20190301065101_20190301155600_0012_A.2C
11	CE4_GRAS_LPR-1_SCI_N_20190301155601_20190302021300_0013_A.2C
12	CE4_GRAS_LPR-1_SCI_N_20190302131101_20190302160000_0016_A.2C
13	CE4_GRAS_LPR-1_SCI_N_20190303052101_20190303100000_0018_A.2C
14	CE4_GRAS_LPR-1_SCI_N_20190310092701_20190311064400_0020_A.2C
15	CE4_GRAS_LPR-1_SCI_N_20190311064401_20190312052700_0021_A.2C
16	CE4_GRAS_LPR-1_SCI_N_20190312052701_20190312110000_0022_A.2C
17	CE4_GRAS_LPR-1_SCI_N_20190329122801_20190331080000_0023_A.2C
18	CE4_GRAS_LPR-1_SCI_N_20190409110001_20190411050000_0032_A.2C
19	CE4_GRAS_LPR-1_SCI_N_20190428060001_20190429153000_0033_A.2C
20	CE4_GRAS_LPR-1_SCI_N_20190430080001_20190501120000_0034_A.2C
21	CE4_GRAS_LPR-1_SCI_N_20190508070001_20190509073000_0036_A.2C
22	CE4_GRAS_LPR-1_SCI_N_20190509080001_20190509110000_0037_A.2C
23	CE4_GRAS_LPR-1_SCI_N_20190528000001_20190528080000_0039_A.2B
24	CE4_GRAS_LPR-1_SCI_N_20190529070001_20190529100000_0040_A.2B

25	CE4_GRAS_LPR-1_SCI_N_20190529100001_20190529120000_0041_A.2B
26	CE4_GRAS_LPR-1_SCI_N_20190530070001_20190530160000_0042_A.2B
27	CE4_GRAS_LPR-1_SCI_N_20190607030001_20190609140000_0043_A.2B
28	CE4_GRAS_LPR-1_SCI_N_20190627040001_20190628070000_0044_A.2B
29	CE4_GRAS_LPR-1_SCI_N_20190706073001_20190707040000_0047_A.2B
30	CE4_GRAS_LPR-1_SCI_N_20190707110001_20190708050000_0048_A.2B
31	CE4_GRAS_LPR-1_SCI_N_20190726000001_20190727080000_0049_A.2B
32	CE4_GRAS_LPR-1_SCI_N_20190728010001_20190729020000_0050_A.2B
33	CE4_GRAS_LPR-1_SCI_N_20190805020001_20190805050000_0052_A.2B
34	CE4_GRAS_LPR-1_SCI_N_20190805050001_20190806120000_0053_A.2B
35	CE4_GRAS_LPR-1_SCI_N_20190825003001_20190825053000_0054_A.2B
36	CE4_GRAS_LPR-1_SCI_N_20190827060001_20190827100000_0060_A.2B
37	CE4_GRAS_LPR-1_SCI_N_20190903020001_20190903123000_0061_A.2B
38	CE4_GRAS_LPR-1_SCI_N_20190904153001_20190905083000_0067_A.2B

Supplementary Notes 1: Geologic settings of Chang'e-4 (CE-4) landing site

Von Kàrmàn's neighboring region is geologically highly complex: the map I-1047[1] and the inset[2], show a superposition of impact morphologies spanning from the pre-Nectarian to the Copernican epochs (Supplementary Figure 1d). Recent remote sensing missions have produced, among others, high-spatial resolution lunar topography maps describing the topography (i.e., SLDEM2015+LOLA[3], Supplementary Figure 1b) and the mass distribution (GRAIL gravity data[4], Supplementary Figure 1c) offering new interpretative elements for reconstructing the evolution of the region.

When looking at complementary data the age relationship between the 'younger' Leibnitz (228 km) and Von Kármán (171 km, VK hereafter) craters is not as clearly defined as it is suggested from photography[5] (Supplementary Figure 1b and 1c): the reshaping of the northern rim section of VK is mostly superficial and due to the deposition of the alleged antipodal surge from the distal Imbrium impact (Ig, I-1047 map, Supplementary Figure 1d) and ejecta from the Finsen crater (Ec). The elevation profile (A-B, Supplementary Figure 1c) helps to highlight the similarities in floor topography shown in Supplementary Figure 1a suggesting a common infill history. However, Van Kármán M (VK-M, 250 km) clearly predates the two major craters in the area. The VK projectile hit the large crater some 90 km north of its center causing a lift of the VK-M crater floor up to 800 m above the regional mare median (Supplementary Figure 1a). Later, the Alder (80 km) impact in the south-east further modified its remnant morphology.

These three impacts, VK, VK-M, and Alder produced a large gravity anomaly bound by the superposition of the three morphologies' crater rims with a diameter of about 265 km (Supplementary Figure 1c). The gravity low typically associated with a complex craters' central region (see Leibnitz's), in this case instead lies across the southern rim of VK stretching to its actual peak center (i.e., topographical high). This observation is relevant because the CE-4 exploration path is located at the border of this area.

The time relation between the smaller craters Davisson (87 km), Alder (80 km) and Finsen (71 km) are reflected on their elevation profiles with the first crater showing an infill profile comparable to the larger craters, in particular to its neighboring Leibnitz (Supplementary Figure 1a). The others instead show similar rim-to-floor heights (~4.7 km, against ~3.7 km for Davisson). The time sequence of these craters is relevant to the interpretation of the stratigraphy at the CE-4 exploration path given that their ejecta would potentially influence the radar signal.

Supplementary Notes 2: The penetrating depth analysis of LPR CH-1

Supplementary Figure 2 is the penetration depth of the LPR is calculated using an approach similar to the correlation coefficient method (CCM) proposed by Xing et al., (2017). The noise and radar echo can be differentiated by the corrections between neighboring data tracks. The detection limit is approached when the correlation reaches a low level, e.g. its absolute value is no larger than 0.5 in this work due to high noise.

Supplementary Notes 3: The estimation of average loss tangent value at CE-4 site

In Xing et al. (2017), they used the following radar equation to estimate the penetrating depth with different loss tangent values.

$$G = \frac{\lambda^2 \zeta_1 \zeta_2 \sigma}{(4\pi)^3 R^4} e^{-4\alpha R}$$

Where, G is the system gain， ζ_1, ζ_2 are the penetrating attenuation between two medias along input and output direction, λ is the wavelength in the medium, σ is the backscatter cross section, e^{-4aR} means the attenuation in the medium, and α is the attenuation rate.

The attenuation rate α is calculated by:

$$\alpha = \frac{2\pi}{\lambda_0}\sqrt{\varepsilon}\left[\frac{1}{2}\left(\sqrt{1+tan^2\delta}-1\right)\right]^{\frac{1}{2}}$$

G = 152 dB, f=60 MHz, ε=6.6 (Li et al. (2017), Xing et al., (2017)). The loss tangent is no larger than 0.005 for a detection limit of 300 m. Based on our calculation, the penetrating depth of CE-4 LPR CH-1 is 330 m, meaning the loss tangent is less than 0.005.

We also estimate the loss tangent values with the method used in Lai et al., (2019), as shown in Figure 2. For R^2 correction, $\tan\delta = 0.0060 \pm 0.0001$; for R^3 correction, $\tan\delta = 0.0051 \pm 0.0001$; For R^4 correction, $\tan\delta = 0.0041 \pm 0.0001$.

Overall, the loss tangent values obtained by both approaches lie within the range of the measured results from the Apollo samples and the LRS estimation results (Ono et al. 2009). The loss tangent obtained from the Xing et al., (2017) is closed to the results from the R^3 correction in the second approach, which considers the case of a rough and planar reflector to calculate the scattering sectional area. The reason is that both methods use the same approximation to model scattering sectional area.

Supplementary Notes 4: The LPR simulation results with different dielectric permittivity and loss tangent values of mare basalt

Supplementary Figure 5 shows the simulation results applying different dielectric permittivity values of mare basalt (6.5 vs. 10) and loss tangent values (0.005 vs. 0.009). First, varied permittivity values result in the difference in apparent depth of the reflectors. For example, the reflector E in Supplementary Figure 5a (ε=6.5) appears earlier than that in Supplementary Figure 5b (ε=10) due to its faster propagation speed. However, we have little knowledge about the detailed stratigraphy of the landing site to determine the permittivity value. Other methods such as the crater geomorphology cannot differentiate the lava flows erupted in different periods to offer references. Another prominent feature we observe is that the reflected signal in the deep section is visible in Supplementary Figure 5b (ε=10), but not in Supplementary Figure 5a (ε=6.5) although the loss tangent values stay the same: this is because the large permittivity value increases the dielectric contrasts between mare basalt and paleoregolith or ejecta and the reflectance upon the interface. Therefore, the echo strength of Supplementary Figure 5b is higher than the one in Supplementary Figure 5a. The loss tangent values we obtained in the paper is under the assumption of ε=6.5. Third, the hyperbola shaped signals in D-E section of Supplementary Figure 5a (ε=6.5) become flat in Supplementary Figure 5b (ε=10). Nonetheless, the shape is also affected by factors with high uncertainties, so we did not infer permittivity values using hyperbola method in this work.

Supplementary Notes 5: Data processing steps of channel one (CH-1) of Chang'E-4 lunar penetrating radar (LPR)

1. Data reading
   LPR data collected by Yutu-2 are classified into different levels. The level 0 data were produced by decoding the raw data transmitted from the lunar rover. Then integration, conversion unsigned integers to signed integers, normalized, removing direct current offset, and adding geometric coordinate, data of level 2B are utilized in this paper. A band-pass filter is applied to 2B data to get 2C data file, which is also used in this work. The file list is given in Table S1.

2. Repetitive data removal
   The rover may stop to perform other scientific measurements when LPR is on, so LPR collected data at same location repetitively. We only keep the average value of the same location and LPR data during the movement. An example is given in Supplementary Figure 6. CE4_GRAS_LPR-1_SCI_N_20190209130801_20190210033400_0009_A.2B data file is used, the

moving part is indicated by the red frame.

3. Arrival time removal
   All the data are corrected to the same time-zero position, so that first arrival times of surface echo are the same across the traces.

4. Data file stitching
   The first nine lunar days of LPR data at the frequency of 60 MHz contain 38 files. We assemble all the moving parts and obtain the radargram shown in Supplementary Figure 7.

5. Band-pass filter
   Use Finite Impulse Response (FIR) filter to reduce noises. Its cut-off frequencies are set as 5MHz, 10MHz, 100MHz, 120MHz.

6. Enhancement of weak signal
   To amplify the subsurface weak signals and show the texture of stratigraphy, AGC (Automatic Gain Control)[10] has been applied. (Figure 1a)

7. Adding position information
   The horizontal position is added to the x-axis, the depth of the y-axis is converted with $\bar{\varepsilon} = 4.5\ (\leq 52\ m)/6.5(> 52\ m)$. The final radargram is shown in Figure 1a.

Supplementary Notes 6: Comparisons of LPR CH-1 signals at CE-3 and CE-4 sites

Li et al. (2017) found that the deep radar features of channel one (CH-1) of CE-3 LPR might represent signal artifacts; therefore, we performed a quantitative analysis from three aspects to evaluate the radar features of the CE-4 LPR identified in our work.

1. The locations of horizontal continuous radar features.

As mentioned by Li et al. (2017), weak continuous signals at various depths of 1100, 2500, 3700, and 5800 ns (Figure 3 in Li et al. (2017)) appear in the LPR results of the ground experiments performed on glacier and artificial lunar soil, which cast doubts on the 3700 and 5800 ns features visible in CE-3 LPR results.
Supplementary Figure 8 shows the CE-4 LPR results, the reflectors identified by us located at depth of (A) 723 ns, (B) 935 ns, (C) 1575 ns, (D) 2097 ns, and (E) 3720 ns. Most do not occur at the depth as revealed by signal noises from the ground tests. Although the 3720 ns is close to the depth of 3700 ns, as one of the suspected system noises of CE-3 and ground test results, their characteristics differ: the ringing noise in the ground test (Supplementary Figure 8) is quite straight and continuous, while several short, less continuous and curved signals appear around the reflector in CE-4

(Supplementary Figure 8). If the feature were caused by system noise, it usually continues to be present from the left to the right in the radar image; however, some linear features in CE-4 are quite short and do not cover the entire roving path.

2. Noise level

The signal-to-noise ratios (SNRs) of CE-3 (Supplementary Figure 9a) and CE-4 (Supplementary Figure 9b) LPR CH-1 data are estimated around 3700 ns, here:

$$\mathrm{SNR} = 10 * \log_{10}\left(\frac{Signal^2}{Noise^2}\right)$$

SNR (CE-3) < 2
SNR (CE-4) $= 13.6$

Li et al. (2017) mention that the signal amplitude level of the suspicious reflector around 3700 ns and 5900 ns of CE-3 results is close to the noise level, while the SNR of the identified reflector of CE-4 at 3720 ns is obviously much higher than CE-3 LPR results.

3. Time-Frequency Analysis Applying S-Transform

The time-frequency analysis was performed with S-transform method, adopted from Li et al., (2017). The CE-3 results (Figure 11(c) in Li et al., (2017)) show that the central frequency (10-15MHz) of 3600ns-4000ns section is well below the originally designated frequency (60 MHz), which also happens in the two ground test cases. In Supplementary Figure 10, the central frequency of CE-4 in 3600 ns- 4000 ns section is 35-45 MHz, different from CE-3 and ground test results.

Supplementary References

1. Stuart-Alexander, D. E. *Geologic map of the central far side of the Moon. US Geological Survey, I-1047* (1978). doi:10.3133/i1047

2. Qiao, L., Ling, Z., Fu, X. & Li, B. Geological characterization of the Chang'e-4 landing area on the lunar farside. *Icarus* **333,** 37–51 (2019).

3. Hon De, R. A. Thickness of the western mare basalts. in *10th Lunar and Planetary Science Conference* (1979).

4. Gong, S. *et al.* Thicknesses of mare basalts on the Moon from gravity and topography. *J. Geophys. Res. Planets* **121,** 854–870 (2016).

5. Budney, C. J. & Lucey, P. G. Basalt thickness in Mare Humorum: The crater excavation method. *J. Geophys. Res. Planets* **103,** 16855–16870 (1998).

6. Cohen, B. A. Support for the Lunar Cataclysm Hypothesis from Lunar Meteorite Impact Melt Ages. *Science (80-.).* **290,** 1754–1756 (2000).

7. Ling, Z. *et al.* Composition, mineralogy and chronology of mare basalts and non-mare materials in Von Kármán crater: Landing site of the Chang'E−4 mission. *Planet. Space Sci.* **179,** 104741 (2019).

8. Huang, J. *et al.* Geological Characteristics of Von Kármán Crater, Northwestern South Pole-Aitken Basin: Chang'E-4 Landing Site Region. *J. Geophys. Res. Planets* **123,** 1684–1700 (2018).

9. Rabiner, L. R. & Gold, B. *Theory and application of digital signal processing.*

(Prentice-Hall Inc., 1975).

10. Rabiner, L. R. & Gold, B. *Theory and application of digital signal processing*. (Prentice-Hall Inc., 1975).

11. Xing, S. G. et al. The penetrating depth analysis of Lunar Penetrating Radar onboard Chang'e-3 rover. *Res. Astron. Astrophys*. 17, (2017).

12. Li, C. *et al.* Pitfalls in GPR Data Interpretation: False Reflectors Detected in Lunar Radar Cross Sections by Chang'e-3. *IEEE Trans. Geosci. Remote Sens.* **56,** 1325–1335 (2018).

13. Ono, T. *et al.* Lunar radar sounder observations of subsurface layers under the nearside maria of the Moon. *Science* **323,** 909–912 (2009).
